# Volatile-Free Total Intravenous Anesthesia for Major Gynecologic Surgery in a Manifesting Female Carrier of Becker Muscular Dystrophy: A Case Report

**DOI:** 10.7759/cureus.113956

**Published:** 2026-08-04

**Authors:** Hend Ibrahim, Ibrahim M Eletreby, Bisan A Sabra, Abdalrahman Alasmar

**Affiliations:** 1 Radiology, National Hepatology and Tropical Medicine Research Institute, Cairo, EGY; 2 Anesthesiology, National Cancer Institute, Cairo, EGY; 3 Medicine and Surgery, Al-Quds University, Gaza, PSE; 4 Medicine, Dar Al-Shifa Hospital, Gaza, PSE

**Keywords:** adult granulosa cell tumor, becker’s muscular dystrophy, neuromuscular diseases, risk of malignant hyperthermia, total intravenous anesthesia (tiva)

## Abstract

Becker muscular dystrophy (BMD) is a rare X-linked dystrophinopathy caused by dystrophin gene (DMD gene) variants, resulting in reduced or dysfunctional dystrophin. Despite a milder course than Duchenne muscular dystrophy, patients face significant perioperative risk from skeletal muscle degeneration, respiratory weakness, cardiomyopathy, and conduction abnormalities. Succinylcholine and volatile anesthetics have been associated with life-threatening hyperkalemia, rhabdomyolysis, and malignant hyperthermia-like reactions in dystrophinopathies. Clinically manifest BMD in females is exceptionally rare due to skewed X-inactivation, and perioperative data in this population remain scarce.

We report a 39-year-old woman with genetically confirmed, clinically manifested BMD who underwent elective left salpingo-oophorectomy with surgical staging for a large adnexal mass. She had severe proximal weakness and was non-ambulatory but had no respiratory, swallowing, or aspiration symptoms. Multidisciplinary preoperative evaluation (neurology, cardiology, hepatology, anesthesiology, gynecologic oncology, intensive care) confirmed preserved cardiac function (ejection fraction >70%), normal conduction, no respiratory compromise evidenced by normal pulmonary function tests, and adequate hepatic reserve.

A trigger-free anesthetic strategy was used: the workstation was prepared to eliminate residual volatile agents, and total intravenous anesthesia was maintained with propofol, dexmedetomidine, and midazolam, avoiding succinylcholine and volatile agents. Reduced-dose atracurium provided neuromuscular blockade, and multimodal opioid-sparing analgesia included bilateral rectus sheath blocks and non-opioid analgesics.

Surgery was uneventful, with no hemodynamic instability, arrhythmias, hyperkalemia, or rhabdomyolysis. The patient was extubated in the operating room after full neuromuscular recovery, then electively admitted to the ICU for observation given the risk of delayed respiratory compromise. Her postoperative course was uncomplicated, with stable cardiorespiratory function and effective analgesia. Histopathology confirmed an adult granulosa cell tumor of the ovary.

This case suggests that major gynecologic surgery can be safely performed in clinically manifest female BMD patients through multidisciplinary planning, trigger-free total intravenous anesthesia, careful neuromuscular blockade, opioid-sparing analgesia, and planned ICU surveillance, offering valuable guidance for this rare, high-risk population.

## Introduction

Becker muscular dystrophy (BMD) is a rare X-linked recessive neuromuscular disorder caused by pathogenic variants in the dystrophin gene (DMD gene), resulting in reduced quantity or abnormal function of dystrophin [1,2]. Although BMD follows a milder and more slowly progressive course than Duchenne muscular dystrophy, affected patients may develop progressive skeletal muscle weakness together with variable cardiac and respiratory involvement, creating significant perioperative challenges that are not always reflected by the degree of functional impairment [1,3]. Respiratory muscle weakness, dilated cardiomyopathy, cardiac conduction abnormalities, and increased susceptibility to anesthesia-related complications may substantially increase perioperative morbidity, necessitating careful preoperative evaluation and individualized anesthetic planning [2,3].

Patients with dystrophinopathies are particularly susceptible to serious anesthesia-related complications following exposure to succinylcholine and, less commonly, volatile anesthetic agents because of the risk of hyperkalemia, anesthesia-induced rhabdomyolysis, and life-threatening cardiac events [4,5]. Consequently, contemporary anesthetic management emphasizes trigger-free total intravenous anesthesia (TIVA), judicious use and monitoring of neuromuscular blocking agents, multimodal opioid-sparing analgesia, and close postoperative respiratory surveillance to minimize perioperative risk [4,6,7].

Manifesting female carriers of Becker muscular dystrophy (BMD) are uncommon. Only a minority of female dystrophinopathy variant carriers develop clinically significant skeletal muscle symptoms, and disease expression is highly variable because of skewed X-chromosome inactivation. This phenotypic variability makes perioperative risk assessment and anesthetic management more challenging than in affected males [8]. As a result, published evidence describing anesthetic management in this population remains limited, particularly in women undergoing major abdominal or gynecologic surgery [9].

We report the successful perioperative management of a woman with genetically confirmed, clinically manifest BMD who underwent elective left salpingo-oophorectomy for a large adnexal mass. Anesthesia was performed using a trigger-free TIVA technique, avoiding both volatile anesthetic agents and succinylcholine, while incorporating regional anesthesia as part of an opioid-sparing multimodal analgesic strategy. Despite uneventful extubation in the operating room, planned postoperative intensive care monitoring was undertaken because of the anticipated risk of delayed respiratory compromise. This case highlights the importance of preoperative assessment, individualized anesthetic decision-making, and proactive postoperative monitoring, and contributes to the limited literature on the perioperative management of manifesting female patients with BMD.

## Case presentation

**Figure 1:**
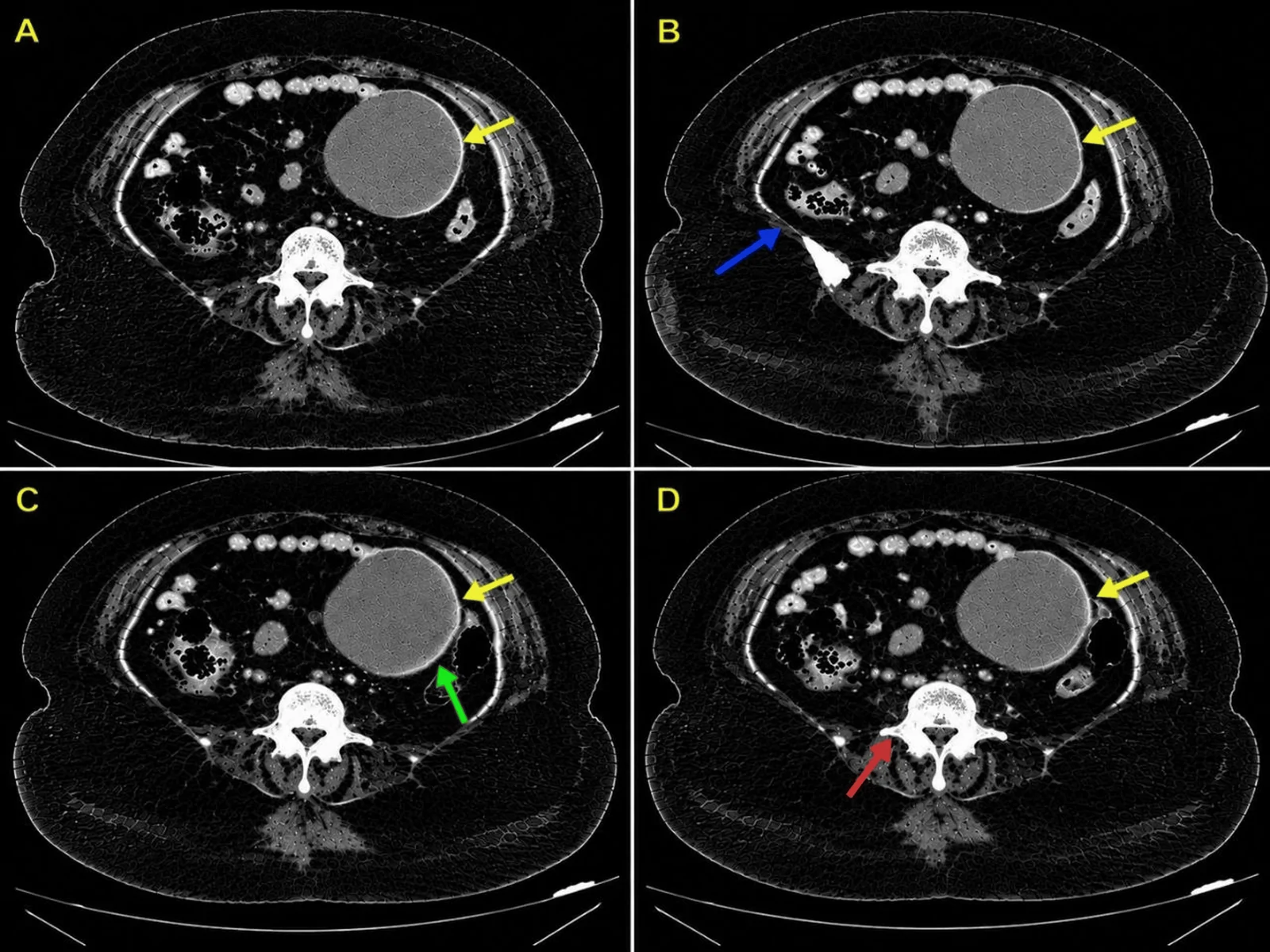
Contrast-enhanced computed tomography (CT) of the abdomen and pelvis. Axial images (A–D) obtained at sequential levels demonstrate a well-circumscribed, predominantly cystic, left adnexal mass measuring approximately 6.0 × 10.0 × 10.5 cm (yellow arrows), arising from the left ovary. The lesion is unilocular with a thin, smooth wall (green arrow) and homogeneous low-attenuation fluid content. There are no internal septations, mural nodules, or solid enhancing components. Mild mass effect on adjacent bowel loops is noted (blue arrow), without evidence of adjacent organ invasion, ascites, or enlarged lymph nodes (red arrow).

A 39-year-old woman (weight 100 kg, height 170 cm, body mass index 34.6 kg/m²) with genetically confirmed Becker muscular dystrophy (BMD) was scheduled for elective left salpingo-oophorectomy with surgical staging for a left adnexal mass measuring 6 × 10 × 10.5 cm (Figure 1). 

The patient was diagnosed with BMD at 13 years of age and had experienced progressive skeletal muscle weakness predominantly affecting the proximal musculature. The disease progressed over the ensuing years, and she became non-ambulatory several years after diagnosis. At the time of presentation, she was unable to walk, climb stairs, or perform antigravity movements of the upper limbs. Motor function was limited to wrist and ankle movements, and she retained the ability to elevate her head. Neurological examination demonstrated severe proximal muscle weakness with relatively preserved distal motor function.

Despite advanced motor disability, she had no clinical symptoms suggestive of respiratory muscle weakness. She denied dyspnea at rest or on exertion prior to losing ambulation. She denied symptoms suggestive of nocturnal hypoventilation. She had no history of non-invasive ventilation, obstructive sleep apnea, respiratory failure, or previous intensive care unit (ICU) admission for respiratory complications. She also had no history of bulbar dysfunction, including dysphagia, dysarthria, or aspiration.

Her family history was significant for BMD affecting two brothers and one sister, with more severe disease reported among the affected female family members. Her medical history included well-controlled hypertension treated with valsartan/hydrochlorothiazide, chronic untreated hepatitis C infection, and persistently elevated liver transaminases (aspartate aminotransferase (AST) 102 U/L and alanine aminotransferase (ALT) 75 U/L), for which she was receiving ursodeoxycholic acid. Four years previously, she had undergone cesarean section under spinal anesthesia without perioperative complications.

Given the underlying dystrophinopathy, a multidisciplinary preoperative assessment was performed involving neurology, cardiology, hepatology, anesthesiology, gynecologic oncology, and intensive care specialists. Neurology recommended comprehensive cardiac evaluation, transthoracic echocardiography, planned postoperative ICU admission, and preparation for the possibility of postoperative mechanical ventilation should respiratory compromise occur. Cardiology deemed the patient fit for surgery. Transthoracic echocardiography demonstrated preserved left ventricular systolic function with an ejection fraction greater than 70%, a left atrial diameter of 35 mm, and mild mitral regurgitation. Electrocardiography demonstrated normal sinus rhythm without conduction abnormalities. Hepatology classified the patient as Child-Pugh class A and cleared her for surgery [10]. Preoperative venous duplex ultrasonography showed no evidence of deep venous thrombosis.

Functional capacity could not be reliably assessed because of severe motor impairment; however, she denied respiratory symptoms and tolerated the supine position without difficulty. Respiratory examination revealed clear bilateral breath sounds. Airway assessment demonstrated a Mallampati class II airway, a thyromental distance greater than 6 cm, and adequate mouth opening [11].

On arrival in the operating room, her blood pressure was 140/80 mmHg and heart rate was 90 beats/min with a regular rhythm. Preoperative liver function tests demonstrated persistently elevated AST (102 U/L) and ALT (75 U/L), consistent with her chronic liver disease. In addition, creatinine was slightly low. The remaining investigations were unremarkable, as shown in the summary Table 1.

**Table 1:** Preoperative laboratory investigations

Investigation	Patient Result	Reference Range
Hemoglobin	12.7 g/dL	12.0–16.0 g/dL
Platelet count	172 × 10⁹/L	150–400 × 10⁹/L
International Normalized Ratio (INR)	1.1	0.8–1.2
Aspartate aminotransferase (AST)	102 U/L ↑	10–40 U/L
Alanine aminotransferase (ALT)	75 U/L ↑	7–56 U/L
Serum creatinine	0.3 mg/dL ↓	0.5–1.1 mg/dL
Blood urea	17 mg/dL	7–20 mg/dL
Sodium (Na⁺)	139 mmol/L	135–145 mmol/L
Potassium (K⁺)	4.3 mmol/L	3.5–5.0 mmol/L
Serum albumin	4.4 g/dL	3.5–5.0 g/dL
Creatine kinase	473 U/L	<200 U/L

Because of the increased risk of anesthesia-related complications associated with dystrophinopathies, a trigger-free general anesthetic was planned, with avoidance of succinylcholine and volatile anesthetic agents. Standard American Society of Anesthesiologists (ASA) monitoring was applied throughout the procedure [12]. Anesthesia was induced with propofol 1 mg/kg, fentanyl 1 µg/kg, and a reduced dose of atracurium 0.3 mg/kg, with all induction drugs dosed according to lean body weight. Anesthesia was maintained using a propofol-based total intravenous anesthesia (TIVA) regimen at 10 mg/kg/h for the first 10 minutes, followed by 8 mg/kg/h for 10 minutes and subsequently 6 mg/kg/h, adjusted according to adjusted body weight. Muscle relaxation was maintained with reduced-dose atracurium (0.05 mg/kg every 30 minutes, based on lean body weight). Dexmedetomidine was administered as a loading dose of 0.5 µg/kg over 20 minutes, followed by a continuous infusion at 0.3 µg/kg/h, with an additional 1-2 mg increments of midazolam administered as required.

A multimodal opioid-sparing analgesic strategy was employed, consisting of intravenous paracetamol (1,000 mg), ketorolac (30 mg), dexamethasone (8 mg), continuous dexmedetomidine infusion, and bilateral rectus sheath nerve blocks. Intraoperatively, the patient received 2,000 mL of Ringer's lactate solution, with an estimated blood loss of 250 mL and a urine output of 650 mL (2.1 mL/kg/h). Mechanical ventilation was provided using volume-controlled ventilation with a tidal volume of 500 mL, respiratory rate of 12 breaths/min, positive end-expiratory pressure (PEEP) of 5 cmH₂O, and an inspired oxygen fraction (FiO₂) of 50%. The total duration of the procedure was three hours. Capnography was monitored throughout the operation with ETCO2 ranging between 29:32 mmHg. Serial potassium levels were monitored (preoperatively: 4.3 mmol/L, on ICU admission: 4.1 mmol/L, 24 hrs after ICU admission: 3.8 mmol/L). Furthermore, serial arterial blood gas (ABG) was performed as shown in Table 2.

**Table 2:** Serial arterial blood gas values The results demonstrate stable acid-base status, adequate oxygenation, and normal lactate throughout the perioperative period. PaO_2_: Partial pressure of oxygen in arterial blood, FiO_2_: Fraction of inspired oxygen, PaCO_2_: Partial pressure of carbon dioxide in arterial blood, HCO_3_^-^: Bicarbonate ion.

Timepoint	pH	PaO₂ (mmHg)	FiO₂	PaO₂/FiO₂ Ratio	PaCO₂ (mmHg)	HCO₃⁻ (mmol/L)	Lactate (mmol/L)
Normal range	7.35–7.45	80–100 (room air)	—	>400	35–45	22–26	0.5–2.0
After induction	7.39	187	50%	374	42	25	1.3
Before extubation	7.36	180	45%	400	46	23	2.1
On ICU admission (room air)	7.34	89	21%	423	45	22	1.6
24 hours after ICU admission (room air)	7.38	86	21%	409	42	24	1.8

The ultrasound-guided bilateral rectus sheath block was performed using an in-plane lateral-to-medial approach with a high-frequency linear transducer (6-15 MHz) to provide postoperative analgesia for the planned midline incision extending from above the umbilicus to the symphysis pubis, corresponding to the T7-T12 dermatomes. A 22-gauge block needle was advanced under continuous ultrasound guidance, and after negative aspiration every 3-5 mL to minimize the risk of inadvertent intravascular injection, 20 mL of 0.25% bupivacaine was injected into the rectus sheath plane on each side (total volume 40 mL), ensuring that the maximum recommended local anaesthetic dose was not exceeded. No block-related complications were observed. The rectus sheath block was incorporated as part of the multimodal analgesic strategy to provide somatic analgesia for the midline abdominal incision; however, it was recognized that this technique does not provide visceral analgesia.

The surgical procedure was completed uneventfully without hemodynamic instability, cardiac arrhythmias, hyperkalemia, metabolic derangements, or clinical evidence of anesthesia-induced rhabdomyolysis. Following reversal of neuromuscular blockade using neostigmine 5 mg and atropine 2 mg, the patient fulfilled standard extubation criteria and was extubated uneventfully in the operating room. In accordance with the multidisciplinary perioperative plan, she was admitted electively to the ICU for postoperative observation because of the anticipated risk of delayed respiratory compromise.

The patient's postoperative course was uneventful, with stable respiratory and cardiovascular status, satisfactory pain control, no neurological deterioration, and no requirement for ventilatory support. Respiratory rate remained stable, ranging from 16 to 20 breaths/min during the 24-hour intensive care unit (ICU) stay. Oxygen saturation was maintained between 96% and 99% on room air throughout the ICU admission. Serum potassium remained within the normal range at 3.8 mmol/L. Creatine kinase (CK) increased from a preoperative baseline of 473 U/L to 842 U/L postoperatively, without associated clinical evidence of rhabdomyolysis or other complications. Body temperature was monitored using an oral thermometer and remained stable, ranging from 37.0°C to 37.6°C during the ICU stay.

Postoperative pain was assessed using the Numerical Rating Scale (NRS). Pain scores ranged from 2 to 3 at rest and 4 to 5 during movement, indicating satisfactory analgesia. No postoperative nausea or vomiting was reported. The patient was monitored in the ICU for 24 hours, followed by transfer to the surgical ward for an additional four days, resulting in a total postoperative hospital stay of five days. She was discharged in stable condition with planned follow-up in the surgical outpatient clinic. A summary of the perioperative management is displayed in Table 3.

**Table 3:** Summary of the perioperative management EF: Ejection fraction, TIVA: Total intravenous anesthesia.

Domain	Finding	Management
Respiratory	No symptoms	ICU observation
Cardiac	EF >70%	Cardiology clearance
Neuromuscular	Severe proximal weakness	Trigger-free TIVA
Analgesia	High respiratory risk	Rectus sheath block + multimodal analgesia
Airway	Mallampati II	Standard laryngoscopy
ICU	High neuromuscular risk	Planned monitoring

## Discussion

Patients with Becker muscular dystrophy (BMD) represent a high-risk population for anesthesia because perioperative morbidity is largely determined by the underlying neuromuscular disease rather than the surgical procedure itself. Progressive skeletal muscle degeneration, variable cardiomyopathy, respiratory muscle involvement, and abnormal responses to anesthetic agents necessitate meticulous perioperative planning [2,3].

Importantly, the degree of perioperative risk may not correlate with the severity of motor disability, as patients with advanced muscle weakness may still have relatively preserved cardiopulmonary function. In the present case, despite severe motor impairment and loss of ambulation, the patient reported no respiratory symptoms. Preoperative pulmonary function testing demonstrated normal lung function (forced vital capacity (FVC) 3.19 L, 119.8% predicted; forced expiratory volume in 1 second (FEV₁) 2.55 L, 112.4% predicted; FEV₁/FVC ratio 79.8%, above the lower limit of normal of 69.46%). These findings did not suggest clinically significant respiratory impairment [2,3].

Avoidance of triggering anesthetic agents remains the cornerstone of anesthetic management in patients with dystrophinopathies [4,6]. Succinylcholine is contraindicated because of the risk of life-threatening hyperkalemia, rhabdomyolysis, and cardiac arrest resulting from muscle membrane instability and upregulation of extrajunctional acetylcholine receptors [4,5]. Although dystrophinopathies are not considered classic malignant hyperthermia disorders, exposure to volatile anesthetic agents has been associated with anesthesia-induced rhabdomyolysis and malignant hyperthermia-like reactions. Consequently, current recommendations favor a trigger-free anesthetic technique using TIVA [4,6].

To minimize the risk of inadvertent exposure to volatile anesthetic agents, the anesthesia workstation was prepared according to trigger-free anesthesia recommendations [6,7]. Anesthesia was delivered using a GE Healthcare anesthesia workstation (Carestation 650 model). To minimize exposure to volatile anesthetic agents, the vaporizers were removed, the CO₂ absorbent was replaced, and the workstation was flushed with a high fresh gas flow of 10L/min for 90 min before induction according to our institutional protocol. Activated charcoal filters were not used, and residual volatile anesthetic concentrations were not measured. A propofol-based TIVA technique was subsequently used throughout the procedure without exposure to inhalational anesthetics. The absence of intraoperative hyperkalemia, rhabdomyolysis, cardiac arrhythmias, hemodynamic instability, or other anesthesia-related complications denotes an uneventful approach in this patient with BMD undergoing general anaesthesia [5,6].

Selection of the neuromuscular blocking agent also warrants careful consideration in patients with BMD [6,13]. Atracurium was selected over rocuronium because our institution lacked routine access to sugammadex and its organ-independent elimination reduced concern regarding altered pharmacokinetics in this patient with chronic liver disease. These pharmacologic properties reduce the risk of prolonged neuromuscular blockade in patients with underlying neuromuscular disease [6,13]. Complete recovery from neuromuscular blockade was confirmed clinically before extubation. In addition to meeting standard extubation criteria, the patient demonstrated sustained head elevation for more than five seconds, adequate spontaneous tidal volumes (>8 mL/kg), sustained hand grip, tongue protrusion on command, intact protective airway reflexes, and full consciousness with appropriate responses to verbal commands. More than one hour had elapsed since the final dose of atracurium. These findings supported safe extubation in the operating room after confirming adequate spontaneous ventilation. Equipment, airway devices, and experienced personnel remained immediately available for reintubation should respiratory compromise have occurred.

Multimodal opioid-sparing analgesia constituted another important component of the anesthetic strategy [14]. Bilateral ultrasound-guided rectus sheath blocks and intravenous non-opioid analgesics provided satisfactory postoperative pain control while minimizing systemic opioid administration. Postoperative pain was assessed using the Numerical Rating Scale (NRS) during the first 24 hours in the intensive care unit. Pain scores ranged from 2 to 3 at rest and 4 to 5 during movement. Analgesia was provided with intravenous paracetamol 1 g every 6-8 hours, which provided adequate postoperative pain control. Reducing opioid requirements is particularly advantageous in patients with neuromuscular disorders because opioid-induced respiratory depression may further increase the risk of postoperative ventilatory compromise [14].

Cardiovascular and respiratory evaluation remain fundamental in patients with dystrophinopathies because cardiomyopathy and respiratory muscle weakness are major determinants of perioperative morbidity and mortality [2,3]. Although our patient demonstrated preserved ventricular function and no evidence of clinically significant respiratory involvement, elective postoperative admission to the intensive care unit was planned because delayed respiratory deterioration and anesthesia-related complications have been described even after an apparently uneventful anesthetic [3,6]. Furthermore, anesthesia-induced rhabdomyolysis and malignant hyperthermia-like reactions may not become clinically apparent until several hours after surgery [4,5]. For this reason, postoperative observation in an intensive care unit staffed by personnel experienced in the recognition and management of airway and neuromuscular emergencies was considered preferable to routine ward care, where immediate intervention may be less readily available. The patient's postoperative course remained uncomplicated, and she required neither ventilatory support nor additional organ support before transfer to the surgical ward after 24 hours.

Final histopathological examination confirmed an adult granulosa cell tumor of the ovary, validating the indication for definitive surgical treatment [15-17]. Although the pathological diagnosis did not influence the anesthetic management, the need for definitive oncologic surgery precluded delaying surgical intervention despite the patient's underlying neuromuscular disorder. This emphasized the importance of selecting an anesthetic strategy that minimized perioperative risk while facilitating timely surgical treatment. The coexistence of a rare ovarian malignancy and clinically manifest Becker muscular dystrophy further underscores the complexity of perioperative decision-making and adds to the uniqueness of this case.

## Conclusions

Becker muscular dystrophy presents unique perioperative anesthetic challenges because of the potential for cardiopulmonary involvement and serious anesthesia-related complications. This case demonstrates that successful perioperative management of a clinically manifest female patient with BMD undergoing major gynecologic surgery can be achieved through comprehensive multidisciplinary assessment, meticulous trigger-free anesthetic planning, careful preparation of the anesthesia workstation, avoidance of succinylcholine and volatile anesthetic agents, appropriate neuromuscular blockade, multimodal opioid-sparing analgesia, and structured postoperative monitoring. Given the rarity of clinically manifest BMD in adult women, this report adds to the limited literature on anesthetic management in this population and supports an individualized, multidisciplinary approach to optimize perioperative safety. Additional well-documented case reports are needed to strengthen the evidence base and further inform perioperative anesthetic management in this uncommon but high-risk patient population. Although the limitations in our study included the lack of neuromuscular blockage monitoring and processed electroencephalography (EEG) in our facility, we highlight the importance of using them in similar cases.
